# Fishmeal supplementation during ovine pregnancy and lactation protects against maternal stress-induced programming of the offspring immune system

**DOI:** 10.1186/s12917-015-0573-8

**Published:** 2015-10-15

**Authors:** Rebecca E. Fisher-Heffernan, Mamun M. Or’Rashid, Ousama AlZahal, Margaret Quinton, Herman J. Boermans, Brian W. McBride, Timothy R. H. Regnault, Niel A. Karrow

**Affiliations:** Department of Animal and Poultry Science, University of Guelph, 50 Stone Road East, Building 70, Guelph, Canada; Department of Biomedical Sciences, University of Guelph, Guelph, ON Canada; Department of Obstetrics and Gynaecology, Children’s Health Research Institute, Lawson Health Research Institute, Western University, London, ON Canada; Department of Physiology and Pharmacology, Western University, London, ON Canada

**Keywords:** Fishmeal, Sheep, Dermal hypersensitivity test, Fetal programming

## Abstract

**Background:**

Prenatally stressed offspring exhibit increased susceptibility to inflammatory disorders due to *in utero* programming. Research into the effects of n-3 PUFAs shows promising results for the treatment and prevention of these disorders. The purpose of this study was to investigate whether maternal fishmeal supplementation during pregnancy and lactation protects against programming of the offspring’s immune response following simulated maternal infection.

**Methods:**

In order to accomplish this, 53 ewes were fed a diet supplemented with fishmeal (FM; rich in n-3 PUFA) or soybean meal (SM; rich in n-6 PUFAs) from day 100 of gestation (gd 100) through lactation. On gd135, half the ewes from each dietary group were challenged with either 1.2 μg/kg *Escherichia coli* lipopolysaccharide (LPS) endotoxin to simulate a bacterial infection, or saline as the control. At 4.5 months of age the offspring’s dermal immune response was assessed by cutaneous hypersensitivity testing with ovalbumin (OVA) and *candida albicans* (CAA) 21 days after sensitization. Skinfold measurements were taken and serum blood samples were also collected to assess the primary and secondary antibody immune response.

**Results:**

Offspring born to SM + LPS mothers had a significantly greater change in skinfold thickness in response to both antigens as well as a greater secondary antibody response to OVA compared to all treatments.

**Conclusions:**

Supplementation during pregnancy with FM appears to protect against adverse fetal programming that may occur during maternal infection and this may reduce the risk of atopic disease later in life.

## Background

Adverse uterine environments caused by maternal stress and infection can alter the planned programming of various tissues, organs and systems of the fetus. When the re-programming of these systems do not match the predicted environment, the offspring may be more susceptible to inflammatory diseases such as atopy, cardiovascular disease and type II diabetes in later life [1–10]. This hypothesis has recently become one of the main focuses in atopic disease research.

With the increase in atopic disorders, such as asthma, food allergies and atopic dermatitis in the Westernized population, it is speculated that genetic predisposition itself cannot be solely responsible, and focus is being placed on *in utero* events and environmental factors that may be playing a contributing role [11–13]. Prenatal stress and the associated rise in glucocorticoids (GCs), as well as the high concentration of pro-inflammatory mediator omega-6 polyunsaturated fatty acid (n-6 PUFA) has been found to be a factor contributing to the susceptibility to atopic diseases by altering the programming of both the immune system and hypothalamic-pituitary-adrenal axis (HPAA) [14, 15]. For example, alterations in the HPAA through fetal programming have been shown to increase the occurrence of respiratory, and skin diseases [16–18]. These alterations in HPAA programming may be responsible for the typical increase in T helper type 2 (Th2) lymphocytes as well as the associated cytokines and chemokines observed in individuals who were prenatally stressed and those with atopic disease [19, 20]. During normal pregnancy the dominant immune response is of Th2 origin and this helps to facilitate maternal tolerance for the fetus. Shortly after parturition the balance between Th2:Th1 is restored. However, in prenatally stressed individuals, it has been suggested that this shift may be delayed, which may increase the susceptibility to atopic diseases [11].

Recent studies suggest that supplementation with omega-3 polyunsaturated fatty acids (n-3 PUFAs) may help to alleviate atopic disorders during both childhood and adulthood [21–23]. Unlike n-6 PUFAs, n-3 PUFAs promote anti-inflammatory mediators and may help protect against inflammatory challenges. For example, n-3 PUFAs have been shown to alter T lymphocyte gene expression profiles by suppressing their differentiation. Their function is also inhibited due to decreased concentrations of cytokines, chemokines and immunoglobulins associated with these responses [24–26]. However, it appears that the timing, type and dosage of n-3 PUFA supplementation may be crucial in the treatment of atopic disease, as various studies have also shown no beneficial affects with supplementation [27, 28]. Previous studies have focused their efforts on postnatal impacts, however the role of n-3 during pregnancy and an activation of protection is ill defined.

Therefore, the purpose of this study was to investigate whether maternal fishmeal (FM) supplementation rich in n-3 PUFA can protect the offspring’s immune system from simulated maternal infection. It was hypothesized that maternal supplementation with n-3 PUFAs would protect the offspring from maternal endotoxin challenge and will decrease the dermal immune response and antibody-specific response to novel antigens. In order to test this objective a sheep model will be used. Sheep are an excellent model for humans as their offspring are a similar size at birth, and their brain development occurs during fetal development.

## Methods

### Ewe parameters and experimental procedures

Fifty-three cross-bred Rideau-Arcott ewes were used in a randomized block design. All animals were housed at the Ontario Ministry of Agriculture, Food and Rural Affairs (OMAFRA) Ponsonby Sheep Research facility. Beginning on day 100 of gestation (gd 100; gestation period ~145 days) ewes were allocated to a diet rich in either fishmeal (FM; high in n-3 PUFA) or soybean meal (SM; high in n-6 PUFA) and maintained on the diet through 50 days of lactation. The SM diet was considered the control diet in this study because this diet is commonly fed to sheep in Ontario, Canada. Ewes were housed individually indoors in an 8′ × 4′ pen and offered feed twice a day at 2.5 % of body weight for a total amount of 2.64 kg of feed/day (0.312 kg supplement, 0.441 kg mixed grain, 0.630 kg chopped hay and 1.261 kg alfalfa pellet) with average feed intake of 2.53 kg of feed/day in the FM group and 2.59 kg of feed/day in the SM group during gestation. During lactation 3.90 kg of feed/day was offered (0.455 kg supplement, 0.652 kg mixed grain, 0.931 kg chopped hay and 1.862 kg alfalfa pellets) with average feed intake of 3.83 kg of feed/day in the FM group and 3.877 kg of feed/day in the SM group. The amount of DHA and EPA fed per day in the FM supplement was 0.85 g/day during gestation and 1.23 g/day during lactation, while that of the SM was 0.10 g/day during gestation and 0.15 g/day during lactation. In comparison the amount of linoleic acid fed per day in the FM supplement was 0.85 g/day during gestation and 1.33 g/day lactation, while 1.24 g/day during gestation and 1.94 g/day during lactation was fed per day in the SM supplement. This resulted in n-6:n-3 ratios of 1.0 for the FM supplement and 13.2 for the SM supplements. Nutrient requirements were based on both the weight and age of the ewes and were calculated from the Cornell Net Carbohydrate and Protein System for sheep (Cornell University, Ithaca, NY). Data from a preliminary trial demonstrated that dietary PUFA concentrations plateau approximately 27 days after the introduction of the dietary supplement [29]. A detailed dietary composition can be found in Tables 1 and 2.

**Table 1:** Diet and nutrient composition (DM basis) for soybean meal (SM) and fishmeal (FM) supplemented diets fed to ewes

Ingredients^a^	Soybean meal (% DM)	Fishmeal (% DM)
Mixed grain (barley, oats, corn)	16.34	16.34
Alfalfa hay	24.72	24.72
Alfalfa pellets	47.26	47.26
Supplement		
Soybean meal	6.62	-
Fishmeal	-	4.73
Feather meal	2.36	1.47
Wheat grain	0.71	2.36
Wheat shorts	-	1.68
Calcium mono phosphate	0.66	0.25
Calcium carbonate	0.57	0.42
Salt	0.42	0.38
Magnesium oxide	0.19	0.23
Mineral and vitamins^b^	0.16	0.16
Chemical composition^c^		
DM (%)	90.7	90.7
CP (N X 6.25)	21.6	21.6
ADF	22.7	22.5
NDF	35.3	36.3
Lignin	4.7	4.8
Crude fat	2.6	2.8
NFC^d^	37.6	37.1
ME^e^(MCAL/kg)	2.1	2.1

*DM* dry matter, *SM* soybean meal, *FM* fishmeal, *NFC* non-fibre carbohydrate

The following are presented as % DM: CP, ADF, NDF, lignin, crude fat and NFC. Adapted from Or-Rashid et al. [52]

^a^The inclusion rate for all dietary ingredeints is presented on a % dry matter basis

^b^Master Feeds Inc., London, Ontario, Canada

^c^All chemical components expressed on a % of dry matter basis except ME

^d^Non-fibre carbohydrates = 100 -[(NDF - neutral detergent insoluble protein) + CP + crude fat + ash]

^e^Calculated using the Cornell Net Carbohydrate and Protein System for Sheep (CNCPS-Sheep v.1.0.21, Cornell University, Ithica, NY, USA) using chemical analysis

**Table 2:** Fatty acid composition of various dietary components: alfalfa pellets, alfalfa hay, mixed grains (barley, oats, and corn), the SM and FM supplements presented as content (g)/100 g

Percent of total fatty acid	Alfalfa pellets	Alfalfa hay	Mixed grain	SM supplement	FM supplement
10:0	0.266	0.000	0.000	0.202	0.142
12:0	0.538	0.328	0.025	0.132	0.117
14:0	1.274	0.592	0.313	0.770	3.049
14:1	0.061	0.000	0.000	0.052	0.070
16:0	19.496	23.932	18.974	16.033	19.768
16:1–9c	0.433	0.365	0.206	2.104	4.940
18:0	3.625	3.071	1.623	4.808	3.665
18:l–9c	3.775	3.126	24.987	25.064	16.206
18:l–11c	0.638	0.821	1.185	1.687	3.057
18:2n-6	20.758	21.322	47.905	26.993	17.197
20:0	1.132	1.775	0.240	0.562	0.454
20:1	0.000	0.000	1.008	0.223	1.425
18:3n-3	44.984	41.920	3.007	18.390	10.868
22:0	1.475	1.256	0.206	0.309	0.236
20:4n-6	0.000	0.000	0.000	0.191	0.729
20:5n-3	0.000	0.000	0.000	0.732	6.096
24:0	1.542	1.493	0.322	0.346	0.192
22:5n-3	0.000	0.000	0.000	0.093	0.713
22:6n3	0.000	0.000	0.000	1.309	11.073

On gd135, half of each the ewes from each dietary treatment group were endotoxin challenged with a 2 ml *i.v* bolus of 1.2 μg/kg body weight of lipopolysaccharide (LPS) from *Escherichia coli* 055:B5 (Sigma-Aldrich, Oakville, Ontario) dissolved in saline, or a 2 ml bolus of saline for control (CON). Health status such as febrile response, respiration rate and haptoglobin concentrations of these ewes were previously reported in Stryker et al. [30]. The treatment groups and animal numbers are as follows SM + LPS (*n* = 12), SM + CON (*n* = 13), FM + LPS (*n* = 14) or FM + CON (*n* = 14).

All block trials were conducted under the strict ethical guidelines set out by the University of Guelph Animal Care Committee in accordance with the Canadian Council of Animal Care (Protocol number 07R052).

### Lamb parameters and experimental procedures

Eighty-nine lambs were born from the 53 ewes [(SM + LPS (*n* = 19); SM + CON (*n* = 21); FM + LPS (*n* = 24); FM + CON (*n* = 25)]. In order to ensure adequate milk supply to the lambs, ewes were allowed to raise a maximum of 2 lambs that remained with their dam until 50 days of age. Once weaned lambs were housed in groups indoors at the OMAFRA Ponsonby General Animal Facility. All lambs were fed the same diet of lamb grower and hay *ad libitum*.

At 4.5 months of age, all lambs were antigen sensitized with two 1 ml *i.m.* injections containing of 0.5 mg/ml of ovalbumin (OVA; Sigma-Aldrich, Oakville, Ontario) or 0.5 mg/ml of *candida albicans* (CAA; Greer Laboratories Inc., Lenoir, North Carolina) dissolved in 0.5 mg/ml Quil-A adjuvant and saline. Ten days following sensitization, lambs received a booster of the same concentration of OVA and CAA. On day 21 post sensitization, lambs underwent *i.d.* cutaneous hypersensitivity testing on both sides of the neck using OVA and CAA at a concentration of 100 μg/50 μl saline, as well as saline as a control. Skinfold measurements were performed using Harpenden Skinfold Calipers (Creative Health Products, Ann Arbor, Michigan) 0, 1, 2, 4, 6, 24, 48, and 72 h post challenge.

### Blood sampling and IgG ELISA

Blood samples were collected on day 0, 10, and 21 days post sensitization to assess the primary and secondary immunoglobulin G (IgG) antibody-mediated immune response to OVA. Serum blood samples were collected via jugular venipuncture using 10 ml clot-activated serum vacutainers and allowed to clot for 30 min at room temperature prior to centrifugation at 2500 rpm for 15 min. Serum was stored at −80 °C until IgG specific antibodies were analyzed.

The OVA specific antibody response was measured using an indirect enzyme-linked immunosorbent assay (ELISA). Plates were coated with 1.4 mg/ml OVA dissolved in carbonate coating buffer and stored at 4 °C for 48 h. Plates were then washed five times with 200 μl of phosphate buffered saline (PBS) + 0.05 % tween 20. Blocking solution (ELISA ultrablock, Serotec, Raleigh, NC) was added to the plate at 200 μl per well and incubated for 1 h at room temperature. Plates were again washed five times and serum samples and reference standard curve samples were added to wells at 100 μl/well and incubated for 2 h. Serum was diluted as follows, day 0 samples were diluted 1/50, day 10 1/500 and day 21 1/4000. The plates were again washed 5 times with 200 μl PBS + 0.05 % tween 20 and conjugate rabbit-anti-sheep IgG antibody was added to the plates at a dilution of 1/4000 and incubated for 1 h. The plates were then washed and alkaline phosphatase yellow liquid substrate system (Sigma-Aldrich, Oakville, ON) was then added to the wells at 80 μl and incubated for 30 min. The plate absorbance was read on Victor Wallac Plate Reader at a wavelength of 405 nm. Intra- and inter- plate coefficients of variation for the ELISA plates were 1.48 and 5.24 % respectively.

### Statistical analysis

Separate analyses were performed on both the OVA and CAA skinfold thickness measurements on the offspring at the test site. All sites were analyzed as randomized complete block designs with subsampling and with repeated measurements over time on each subsample using the same model. Measurements of OVA and CAA skinfold thickness were performed on a log scale and measurements at 1, 2, 4, 6, 24, 48, 72 h were expressed as differences from time 0 measurements. The model accounted for the 7 blocks, 53 ewes and 89 lambs as random effects and the 2 diets (fishmeal versus soymeal) and 2 challenges (LPS versus control) plus their interaction, the sex of the offspring, time and interactions among time, challenge and diet as fixed effects. The number of offspring born per ewe was also included as a covariate to account for any *in utero* effects. The mixed model procedure from SAS (version 9.4) was used to perform the analysis. Repeated measurements over time for each offspring were handled according to the approach given by Wang and Goonewardene (2004) which recommends using the best-fitting (co)variance structure over time [31]. The Akaike criterion was used to determine the appropriate structure for these analyses which was unstructured for both the CAA and OVA saline sites, Toeplitz for the CAA test site and heterogeneous autocorrelation for the OVA test site. Linear and quadratic orthogonal polynomial contrasts over time and interactions of these with the diets and challenges were used to assess differences in the changes of skinfold thickness over time among the diets and challenges. The mixed model procedure from SAS (version 9.2) was also used to analyze repeated IgG measurements over time for each offspring per ewe. Significant differences over time were reported at a *P*-value < 0.05 and trends over time were indicated by *P*-values ranging from 0.06 to 0.10. Residual plots were examined for all analyses, and showed no evidence of variance heterogeneity.

## Results

All lambs survived this study with no significant treatment differences in body weight. The plasma n-3 PUFA concentrations at weaning (50 days of age), where the least square means of EPA concentrations were 1.33 for FM and 1.109 for SM offspring and DHA concentrations were 1.72 for FM and 1.53 for SM offspring. At the time of hypersensitivity testing (135 days of age) there was no difference in plasma n-3 concentrations where EPA concentrations were 0.528 for FM and 0.520 for SM offspring and DHA concentrations were 1.16 for FM and 1.26 for SM offspring [32].

### Dermal hypersensitivity response to OVA and CAA Antigens

**Fig. 1:**
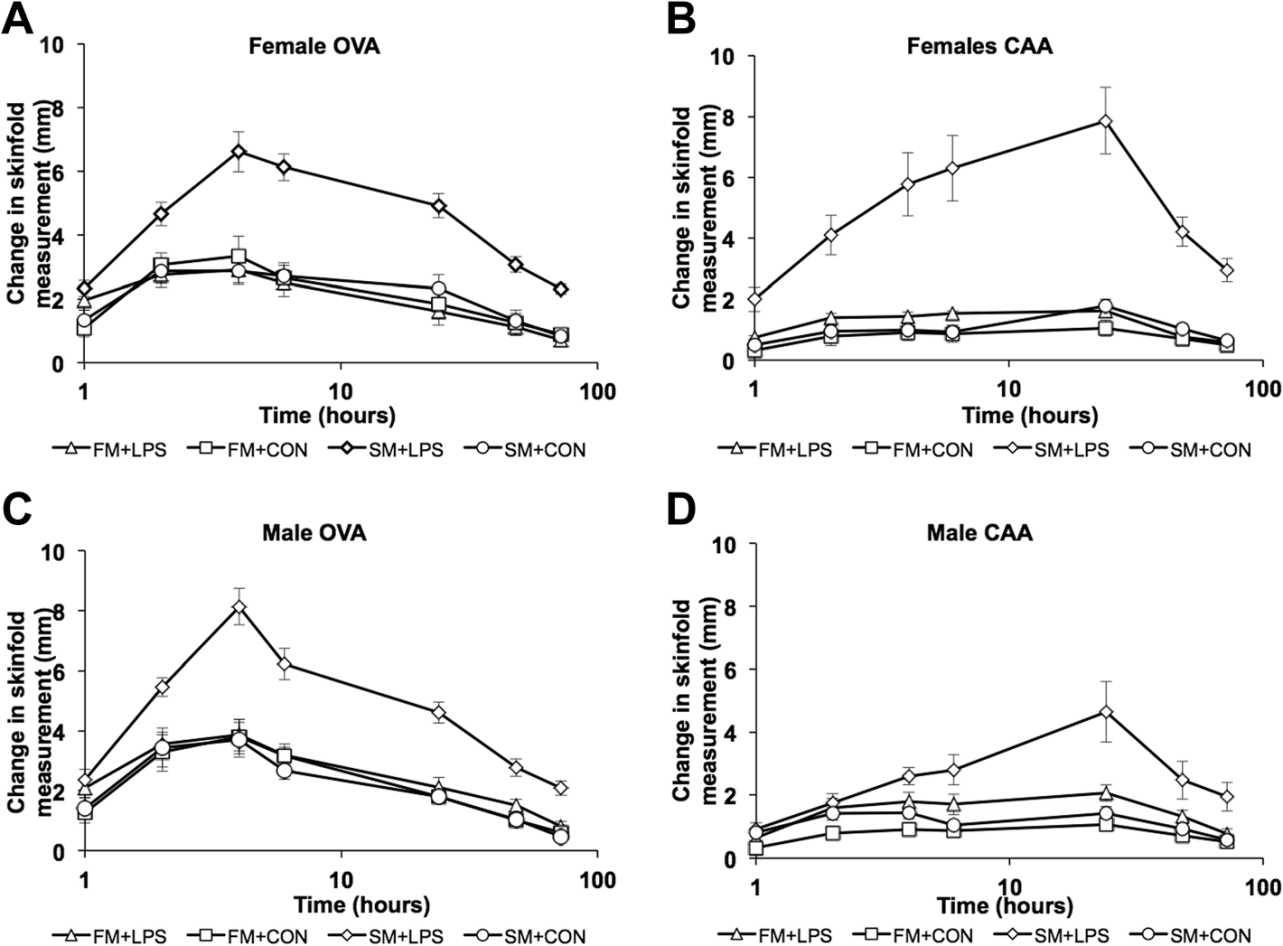
Offspring change in skinfold thickness following cutaneous hypersensitivity test with OVA and CAA. This figure depicts offspring born to mothers supplemented with fishmeal and challenged with saline (FM + CON) or endotoxin (FM + LPS), or offspring born to mothers supplemented with soybean meal and challenged with saline (SM + CON) or endotoxin (SM + LPS). The lettering in the graph represent the specific gender response the antigen; **a** represents the response of female offspring to OVA antigen, **b** represents the response of female offspring to CAA antigen, **c** represents the response of male offspring to OVA antigen and **d** represents the response of male offspring to CAA antigen. Data are reported as least squared means ± SE

All lambs responded to both OVA and CAA dermal hypersensitivity challenge with a significant increase in skinfold thickness represented by both linear and quadratic over time (*P* < 0.05, Fig. 1). Offspring born to SM + LPS mothers demonstrated the greatest increase in skinfold thickness trend to both antigens compared to their control counterparts (SM + CON) as well as both FM treatment groups (FM + LPS, FM + CON, *P* < 0.05; Fig. 1). There was no difference in contrasts for skinfold thickness trends to either OVA or CAA between FM treatment groups (FM + LPS, FM + CON; *P* > 0.05; Fig. 1). There was also a three-way interaction of diet by treatment by quadratic time between the FM + LPS lambs and the SM + LPS lambs to the CAA antigen (*P* < 0.05; Fig. 1 b, d). Therefore, quadratic time trends differed between dietary groups but also between treatment groups.

Linear and quadratic sex difference trends were also observed following both CAA and OVA dermal sensitivity challenge. The diet by treatment by sex contrast demonstrated an increase in skinfold thickness trends, which was greater in female and male offspring born to SM + LPS dams as compared to all other treatment groups within the same sex (*P* < 0.05; Fig. 1). There was also a significant difference in the trends over time with female and male FM + LPS lambs having a slightly greater change in skinfold thickness to the CAA antigen compared to the FM + CON lambs of the same sex (*P* < 0.05; Fig. 1 b, d). This trend was not present at the OVA test site over time in the FM offspring in which both groups responded slightly to the same degree in both sexes (Fig. 1 a, c). A sex difference trend was observed between female and male offspring born to SM + LPS dams over time during the CAA challenge. Quadratic trends demonstrated that SM + LPS female offspring had a greater skinfold thickness response over time to CAA antigen compared to male offspring (*P* < 0.05; Fig. 1 b, d). There were no differences over time observed between male and female offspring born to FM + LPS or FM + CON dams (*P* > 0.05).

### Serum OVA-specific IgG response

**Fig. 2:**
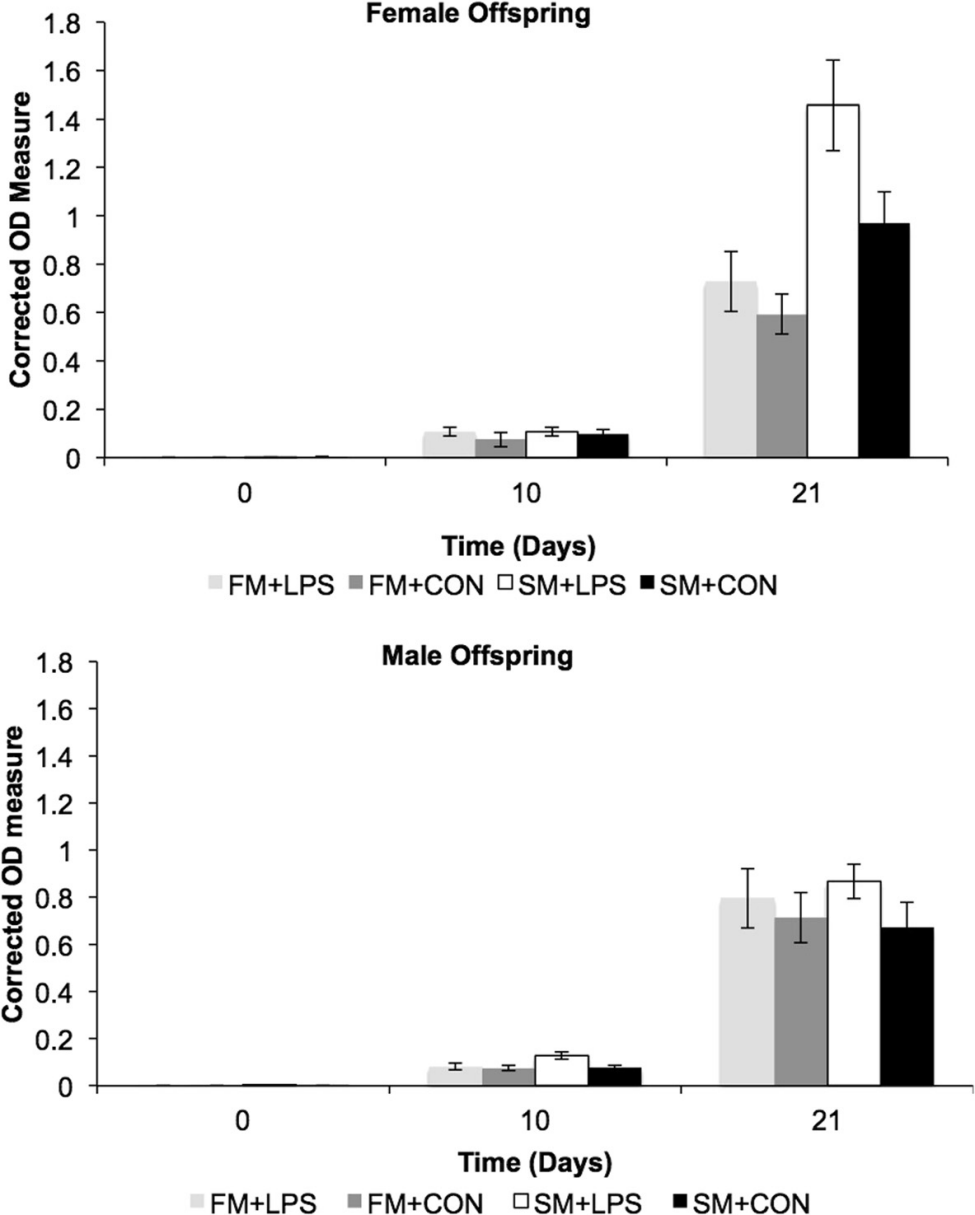
Offspring change in OVA specific IgG response during the primary (d 10) and secondary (d 21) immune response. This figure depicts offspring born to mothers supplemented with fishmeal and challenged with saline (FM + CON) or endotoxin (FM + LPS), or offspring born to mothers supplemented with soybean meal and challenged with saline (SM + CON) or endotoxin (SM + LPS). Data are reported as least squared means ± SE

There was an increase in OVA IgG concentrations over time across all treatment groups demonstrating that the inoculation protocol worked to induce both a primary and secondary IgG immune response (*P* < 0.05; Fig. 2). Offspring from SM + LPS mothers had an increased OVA- specific IgG response during both the primary (d 10) and secondary (d 21) immune response compared to all other treatment groups (*P* < 0.05). Unlike the SM treatment groups, a difference was not observed between offspring born to FM + LPS or FM + CON mothers.

Sex differences were only observed for the secondary IgG immune response. Female offspring from SM + LPS mothers had a greater secondary IgG response compared to their male counterparts (*P* < 0.05; Fig. 2). This trend was not observed in any of the other treatment groups. Additionally, SM + LPS female offspring had a greater concentration of OVA-specific IgG antibodies compared to the female offspring born to both SM + CON and FM + LPS mothers. This response was not observed between male offspring. However, SM + LPS male offspring tended to have a greater IgG response following the primary immune response compared to SM + CON male offspring (*P* < 0.10).

## Discussion

Maternal infection during pregnancy has been shown to increase the offspring’s susceptibility to atopic disease [1]. Recent studies have shown that n-3 PUFA supplementation may provide protection from atopic disease because of its immunomodulatory properties [33–35]. This study investigated whether maternal supplementation with FM (high in n-3) could protect the offspring’s immune system from maternal stress caused by a simulated bacterial infection using endotoxin. The offspring’s immune response was assessed by cutaneous hypersensitivity tests to novel antigens OVA and CAA, as well as the IgG response to OVA. All offspring responded to both antigens with an increase in skinfold thickness, and a primary and secondary response to the OVA antigen. As expected offspring born to mothers supplemented with SM and challenged with endotoxin had the greatest secondary antibody response to OVA antigen as well as the greatest increase in skinfold thickness to both OVA and CAA compared to all other treatment groups. Other studies have demonstrated alterations in both the dermal hypersensitivity response and Ab-mediated response but this was during n-3 PUFA supplementation. One study showed that fish oil supplemented cats had a significantly lower skinfold thickness response to an intradermal injection of histamine compared to the control group [36]. While Lauritzen et al. [37], demonstrated that fish oil supplementation to mouse dams decreased their offspring’s IgG1 antibody response to OVA compared to dams consuming an n-3 PUFA deficient diet or a linseed diet (rich in n-6 PUFA) [37]. The IgE antibody response to OVA has also been shown to be decreased following supplementation with fish oil in mice [38]. Additionally, many studies looking at atopic dermatitis and allergies in humans have also showed improvement in the disorder following supplementation with n-3 PUFAs [28, 39, 40]. Therefore, it appears that n-3 PUFA supplementation may improve atopic disease, however, further research is required to determine the proper dietary inclusion level as well as timing of treatment, as multiple studies that have demonstrated no differences or even increases in both the skinfold thickness and the Ab response with n-3 PUFAs [41, 42]. One should also note that FM and fish sources of n-3 PUFAs might also contain various other fatty acids and amino acids that could also influence the immune response of the offspring.

When comparing to the above-mentioned studies, this is the first study to investigate whether FM supplementation during gestation and lactation can protect the offspring from programming following simulated maternal infection during late gestation. This study is different from others as supplementation with n-3 PUFAs occurred solely through the dam and the offspring n-3 PUFA concentrations were not different across treatments at the time of challenge with OVA and CAA. n-3 PUFA EPA enrichment was observed at 50 days of age with greater concentrations of EPA in the plasma of offspring from FM supplemented dams demonstrating that maternal supplementation during gestation and lactation does in fact cause enrichment in the lambs. However, these differences were small and warrant further investigation into PUFA partitioning in the body to assess whether greater enrichment occurred in other tissues or organs. Nonetheless this suggests that n-3 PUFA supplementation during pregnancy could help reduced the risk of topic disease later in life as well as reducing the risk of infection-induced programming.

Alterations in the ratio of Th1:Th2 lymphocytes and associated mediators have been reported as a factor in the development of atopic disease and it has been suggested that n-3 PUFAs may be acting in a way to restore the balance. For example, various animal models have demonstrated that maternal supplementation with n-3 PUFAs may reverse the skewed Th2 biased response as they have been shown to promote a Th1 population with the increase in IL-2 and IFNγ [25, 43]. This shift in the Th population may explain the differences observed in the skinfold thickness response and Ab-response to OVA in the current study as OVA is typically thought of as a Th2 antigen. However, it does account for the observed decrease in the skinfold thickness response to the CAA, a known Th1 antigen. Therefore, this suggests that a different mechanism may be at work.

Recently, T regulatory cells (T regs) have been shown to play a role in the reduction of atopic disease. T regs are found in the lymph nodes, spleen, and peripheral blood, help to maintain tolerance again allergens [44]. T regs act to suppress the proliferation of Th1 and Th2 lymphocytes as well as their function [44, 45]. It was previously suggested that pregnancy hormones supported a Th2 dominant immune profile, however, new evidence suggests that Tregs induce maternal tolerance to the fetus and help to maintain a successful pregnancy [46]. It has also been shown that maternal stress can reduce the number of T regs at the maternal-fetal interface and this may influence programming of the fetal immune system to one that is less tolerant to novel antigens [11, 47, 48]. Interestingly, supplementation with both DHA and EPA increases the expression of IL-10 and TGF-β as well as Foxp3, a transcription factor required for the production and function of T regs [49]. With this in mind it is speculated that n-3 PUFAs suppress the Th1 and Th2 response by up-regulating T regs and T reg mediators. This may also explain the observed hypersensitivity and Ab suppression in the FM + LPS from the present study offspring and not the SM + LPS offspring to both OVA and CAA antigens, although further research is needed to validate this hypothesis.

Sex differences were also observed in this study between male and female offspring born to SM + LPS mothers. To our knowledge this is the first study to investigate the sex effects of offspring born to mothers supplemented with PUFAs and challenged with endotoxin. It was observed that female offspring had a greater skinfold measurement to the CAA antigen compared to the male offspring. Female offspring also had a greater IgG response to OVA antigen compared to their male counterparts. There was no difference between FM groups observed. This is not surprising, as females have typically demonstrated greater antibody responses to allergens and vaccine. For example, female mice exposed to environmental tobacco smoke, which is known to increase allergic sensitization, and then sensitized with OVA demonstrated greater OVA specific IgG1 and IgE in their serum compared to males [50, 51]. Although females in this study demonstrated the greatest increase in OVA specific IgG, one cannot conclude that the males were not also susceptible to this type of programming, as the male offspring born to SM + LPS mothers also demonstrated great differences in the skinfold measurements to both antigens. This emphasizes the fact that the timing and type of stressor as well as species differences may need to be taken into account for when using animal models. Based on these results of this study, however, it appears that maternal supplementation with FM helps to counteract the *in utero* programming of offspring’s risk of allergic disease later in life regardless of sex.

## Conclusions

Overall this study has provided insight into the potential benefits of supplementing maternal diets with FM to reduce the risk of atopy. It is apparent that FM provides a protective effect from maternal endotoxin challenge during late gestation as indicated by the decrease in offspring change in skinfold thickness in response to OVA and CAA as well as a decreased IgG response to OVA in female offspring. Future studies should focus on the immune and epigenetic mechanisms involved in this protective effect.

## Abbreviations

CAA: *Candida Albicans*
CON: Control treatment group
FM: Fishmeal
HPAA: Hypothalamic-pituitary-adrenal axis
GC: Glucocorticoid
LPS: Lipopolysaccharide
n-3 PUFA: Omega-3 polyunsaturated fatty acid
n-6 PUFA: Omega-6 polyunsaturated fatty acid
OVA: Ovalbumin
SM: Soybean meal
